# Analysis of Essential Liver Anatomy for Anatomical Segmentectomy 8: A Proposal for the Hybrid Glissonean Approach

**DOI:** 10.1002/wjs.70129

**Published:** 2025-10-17

**Authors:** Yosuke Uematsu, Yuta Abe, Yutaka Nakano, Minoru Kitago, Yasushi Hasegawa, Shutaro Hori, Masayuki Tanaka, Minoru Yamada, Masahiro Jinzaki, Yuko Kitagawa

**Affiliations:** ^1^ Department of Surgery Keio University School of Medicine Tokyo Japan; ^2^ Department of Radiology Keio University School of Medicine Tokyo Japan

**Keywords:** Glissonean pedicle, hepatectomy, hepatic veins, minimally invasive surgical procedures, robotic surgical procedures, segmentectomy

## Abstract

**Background:**

Anatomical segmentectomy 8 is challenging owing to the complex branching patterns and position of the Glissonean pedicle of segment 8 (G8) root. The hepatic vein‐guided approach and extrahepatic Glissonean approach are essential for accurate G8 root identification. We evaluated the key anatomical factors for precise G8 root transection and proposed the hybrid Glissonean approach (HYBA) as a potential surgical strategy.

**Methods:**

Using high‐quality multidetector‐row computed tomography (CT) images from 73 living liver donor candidates, we assessed the position of the segment 8 portal vein (P8) root relative to the middle hepatic vein (MHV), intersegmental vein between segments 5 and 8 (IV5/8), and hepatic hilum. Distances from the MHV–IV5/8 confluence and alignment with the MHV/right hepatic vein (RHV) plane were also analyzed.

**Results:**

IV5/8 was not identified on CT in 9.6% of cases. In 61.6% of cases, the P8 root aligned with the MHV–RHV plane. Additionally, in 53.4% of cases, the shortest distance from the P8 root to the MHV matched the MHV–IV5/8 confluence. The distance from the MHV–IV5/8 confluence to the P8 root (5.8–32.5 mm), the first branch of the right anterior portal vein to the P8 root (3.9–39.4 mm), and the shortest distance from the MHV to the P8 root (5.1–28.9 mm) showed significant variation.

**Conclusions:**

This study highlights anatomical variations in the positional relationships of the G8 root with key landmarks (MHV, RHV, IV5/8, and hepatic hilum). Preoperative evaluation of these factors is crucial for selecting the optimal approach, and HYBA has been proposed as a viable option.

## Graphical abstract

Anatomical segmentectomy 8 is challenging owing to the complex branching patterns and position of the Glissonean pedicle of segment 8 (G8) root. The hepatic vein‐guided approach and extrahepatic Glissonean approach are essential for accurate G8 root identification. We evaluated the key anatomical factors for precise G8 root transection and proposed the hybrid Glissonean approach (HYBA) as a potential surgical strategy. Using high‐quality multidetector‐row computed tomography (CT) images from 73 living liver donor candidates, we assessed the position of the segment 8 portal vein (P8) root relative to the middle hepatic vein (MHV), intersegmental vein between segments 5 and 8 (IV5/8), and hepatic hilum. Distances from the MHV–IV5/8 confluence and alignment with the MHV/right hepatic vein (RHV) plane were also analyzed.

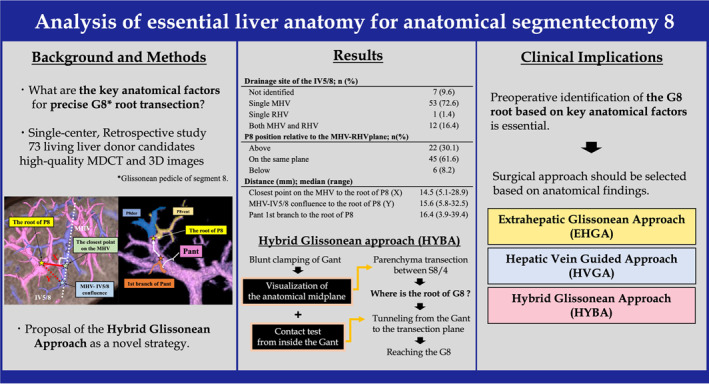

## 1 Introduction

Anatomical liver resection is the complete removal of the liver parenchyma within the portal territory [1]. In anatomical segmentectomy 8, transecting the root of the Glissonean pedicle of segment 8 (G8) and completely resecting segment 8 (S8) are essential. However, this procedure remains challenging owing to variations in the branching pattern [2] and height of the G8. Therefore, preoperative simulation and intraoperative recognition of anatomical landmarks are important for accurately identifying the G8 root [3, 4, 5]. The following two primary techniques are commonly used: the hepatic vein‐guided approach (HVGA) [6, 7] and the extrahepatic Glissonean approach (EHGA) [8, 9].

In HVGA, the liver parenchyma is transected along the middle hepatic vein (MHV) and the intersegmental veins between segments 5 and 8 (IV5/8) using intraoperative ultrasonography. The right side of the MHV and the confluence of IV5/8 with the MHV are used as anatomical landmarks to reach the root of G8 [6, 10, 11]. In EHGA, the anterior Glissonean pedicle (Gant) is exposed from the hepatic hilum to access the G8 root. Depending on the branching pattern, deeper G8 branches may require additional taping of G5 [12]. Although both approaches are useful, accessing the G8 root is difficult in some cases. HVGA is limited when the G8 root is distant from the MHV, such as when the G8 root does not align with the MHV‐right hepatic vein (RHV) plane, when IV5/8 is absent or extremely thin, or when the distance from the MHV‐IV5/8 confluence to the G8 root is particularly long. EHGA becomes difficult when the G8 root is located far from the hepatic hilum, requiring extensive dissection around the Glissonean pedicle.

Although these challenges have been recognized, the anatomical reasons for the difficulty in accessing the G8 root and the frequency of these challenges have not been clearly investigated. We aimed to identify key anatomical factors related to segmentectomy 8 using high‐resolution multidetector‐row computed tomography (MDCT) images from living liver donor candidates. Based on these findings, we propose the hybrid Glissonean approach (HYBA) as a potential option in cases where conventional approaches are limited by anatomical variations.

## 2 Methods

### 2.1 Image Acquisition and Three‐Dimensional (3D) Reconstruction

This retrospective study analyzed donor candidates for living‐donor liver transplantation who underwent high‐quality MDCT at Keio University Hospital between April 2019 and August 2023. Seventy‐three participants (42 men [57.5%] and 31 women [42.5%]) with a median age of 37 years (range: 20–63 years) were included. This study was approved by the Ethics Committee of Keio University School of Medicine (approval number: 20120443) and was conducted in accordance with the Declaration of Helsinki (1975 and its later revisions). The requirement for informed consent was waived due to the retrospective study design.

MDCT examinations were conducted using a 256‐row CT scanner (Revolution CT; GE Healthcare, Chicago, USA) with the following scan parameters: tube voltage, 120 kV; tube current, 10–440 mA (modulated according to each participant's body habitus using the auto mA technique with a noise index of 12 at 5 mm section thickness with filtered back projection); gantry rotation time, 0.5 s; detector collimation, 0.625 × 64 mm; and pitch, 1.375. Iodinated contrast medium, dosed according to participants' body weight (600 mg iodine/kg), was intravenously administered at a fixed injection duration of 25 s using an automatic contrast injector (Dual Shot GX7, Nemoto Kyorindo, Tokyo, Japan). Scans were performed sequentially in the hepatic arterial, portal venous, and hepatic venous phases (with minimum delay times of 20 and 85 s, respectively, after reaching the 150 HU threshold in the abdominal aorta) using bolus tracking. Reconstructed images were processed using a hybrid iterative reconstruction algorithm, with a section thickness of 0.625 mm and 0% overlap to ensure high‐quality CT images [13].

The MDCT datasets were transferred to a dedicated postprocessing workstation (Synapse VINCENT; Fujifilm, Tokyo, Japan), where liver parenchyma images were semi‐automatically extracted from consecutive MDCT images. 3D images of the portal and hepatic veins were generated from the portal or hepatic venous phase data using the automatic algorithm of the software. These extracted portal and hepatic vein images were superimposed, and the 3D images were displayed from multiple angles on the workstation. The straight‐line distances were measured by overlaying various MDCT planes onto the 3D images.

### 2.2 Anatomical Analysis

**FIGURE 1:**
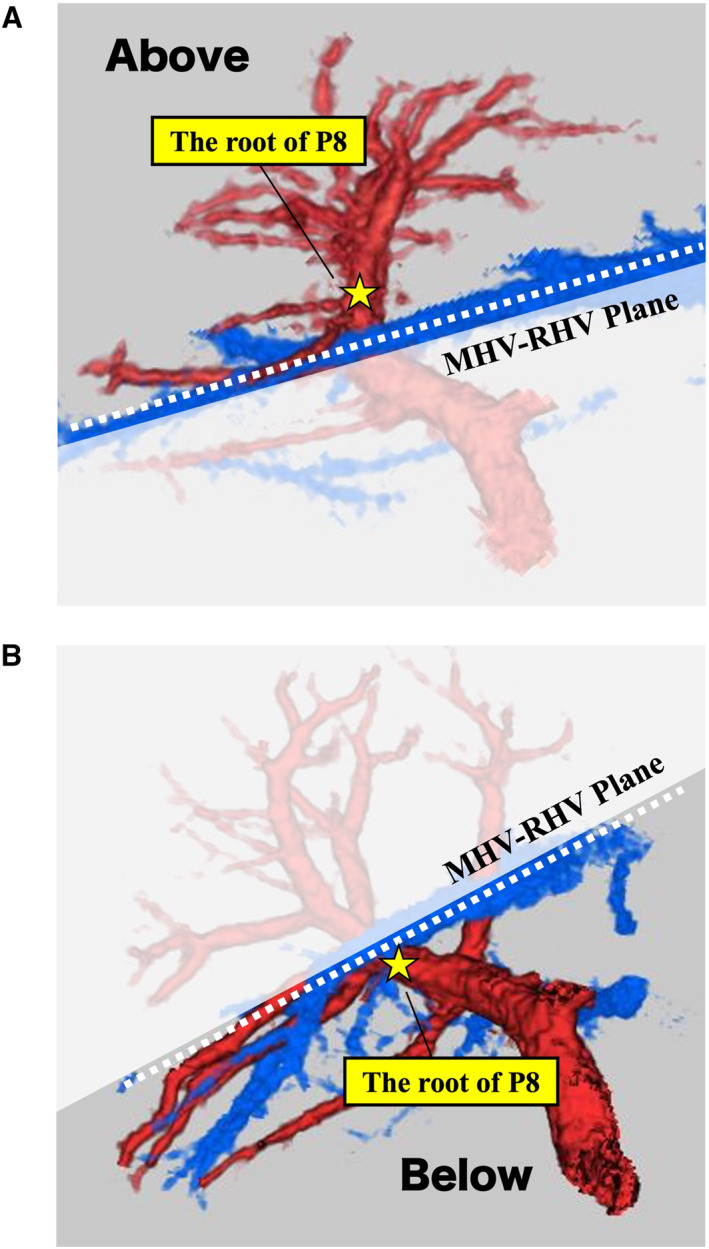
Position of the P8 root relative to the MHV–RHV plane. (A) Classification of the P8 root as “above” when it is located ventral to the MHV–RHV plane. (B) Classification of the P8 root as “below” when it is located dorsal to the MHV–RHV plane. MHV, middle hepatic vein; P8, portal vein of segment 8; RHV, right hepatic vein.

**FIGURE 2:**
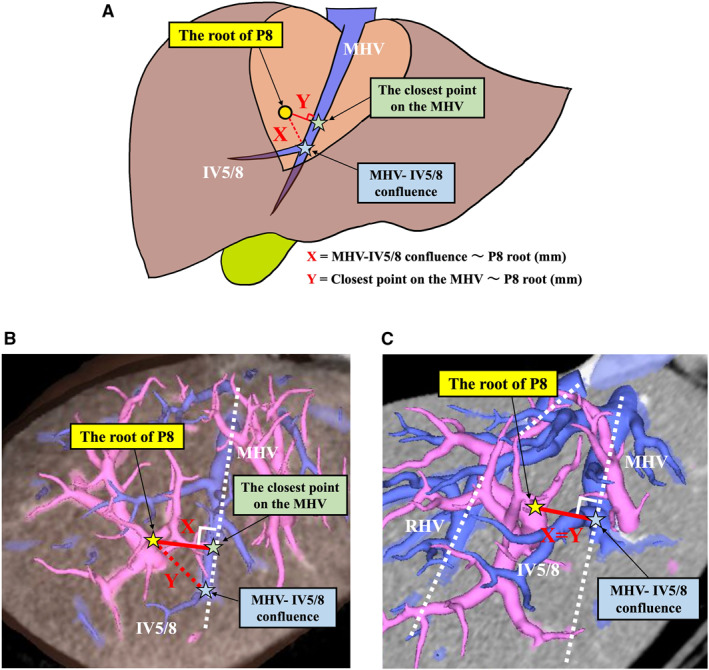
Measuring the distance between the P8 root and the MHV–IV5/8 confluence, as well as the closest point of the MHV. (A) The distance between the closest point of the MHV and the P8 root is defined as “*X*,” and the distance between the MHV–IV5/8 confluence and the P8 root is defined as “*Y*.” (B) The distances (mm) were measured using CT cross‐sections overlaid on 3D simulation images. (C) The shortest distance from the P8 root to the MHV matched the MHV–IV5/8 confluence (*X* = *Y*). IV5/8, intersegmental vein between segments 5 and 8; MHV, middle hepatic vein; P8, portal vein of segment 8.

**FIGURE 3:**
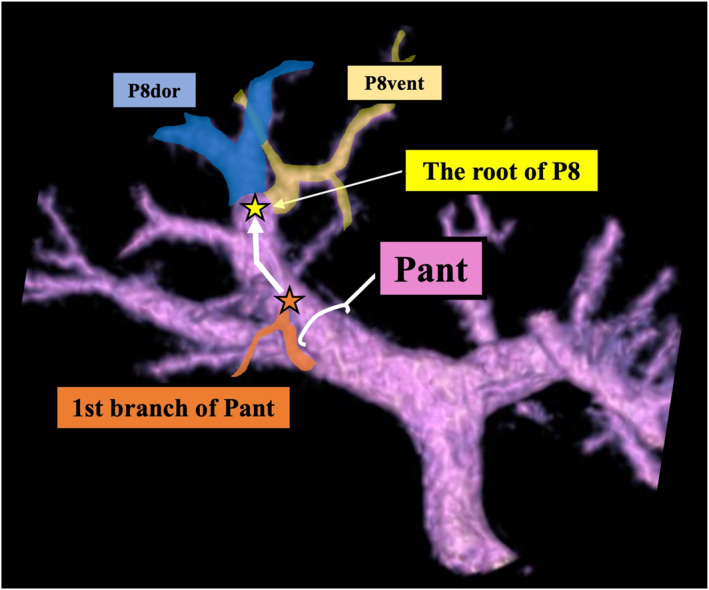
Distance from the first branch of the Pant to the P8 root. The distance (mm) from the first branch of the Pant to the P8 root was measured using 3D simulation images. MHV, middle hepatic vein; P8, portal vein of segment 8; P8 dor, dorsal branch of P8; P8 vent, ventral branch of P8; Pant, right anterior portal vein.

Anatomical factors relevant to anatomical segmentectomy 8 were analyzed, focusing on the following aspects: the branching pattern of the right anterior portal vein (Pant), the drainage site of the intersegmental vein between segments 5 and 8 (IV5/8), the position of the portal vein of segment 8 (P8) root relative to the MHV–RHV plane (Figure 1A,B), the distance between the MHV–IV5/8 confluence and the root of P8 (Figure 2A,B), the shortest distance between the MHV and the root of P8 (Figure 2A,B), and the distance from the first branch of the Pant to the root of P8 (Figure 3).

The branching patterns of the Pant were classified into the following three categories (types): cranio‐caudal, ventro‐dorsal, and trifurcation, based on a previous report [2]. Cases that did not fit these categories were considered “unclassifiable.” To evaluate the ramification pattern of the IV5/8, the portal venous territory was analyzed using region‐growing techniques. The presence of IV5/8 was evaluated, and if present, we determined whether it drained into the MHV, RHV, or both [14]. To evaluate the position of P8 relative to the MHV–RHV plane, 3D images and CT cross‐sections that aligned with the MHV–RHV plane were overlaid. The position of the P8 root relative to the MHV–RHV plane was categorized as “above,” “on the same plane,” or “below” (Figure 1A,B). The shortest distance (mm) between the MHV and the root of P8, defined as “*X*,” and the distance (mm) between the MHV–IV5/8 confluence and the root of P8, defined as “*Y*,” were measured (Figure 2A). These measurements were evaluated by superimposing CT cross‐sections on 3D simulation images, aligning the root of P8, MHV, and MHV–IV5/8 confluence on the same plane (Figure 2B). The frequency of cases where the shortest distance from the P8 root to the MHV matched the MHV–IV5/8 confluence (*X* = *Y*) was also assessed (Figure 2C). Additionally, to evaluate the distance from the hepatic hilum to the P8 root, the distance (mm) from the first branch of the Pant, as identifiable on CT, to the P8 root was measured using 3D simulation images (Figure 3). The exact entry point of the Gant into the liver cannot be reliably defined on CT because the branching points of the Glissonean sheath and its contained structures (including the portal vein) generally do not coincide, particularly in the proximal portion. Because the first small branch from the Pant enters the liver parenchyma, it is located near the hepatic hilum, slightly distal to the Gant root. Therefore, we considered it to reflect the Gant root position more accurately and adopted it in our methodology. The P8 root chosen for this measurement is the one closest to the midplane, which is targeted in HVGA.

### 2.3 Statistical Analyses

Continuous variables are presented as medians with ranges and were compared using the Mann–Whitney *U* test. Categorical variables were compared using the chi‐square test. All statistical analyses were performed using JMP Pro 17.0.0 (SAS Institute Inc., Cary, NC, USA).

## 3 Results

**FIGURE 4:**
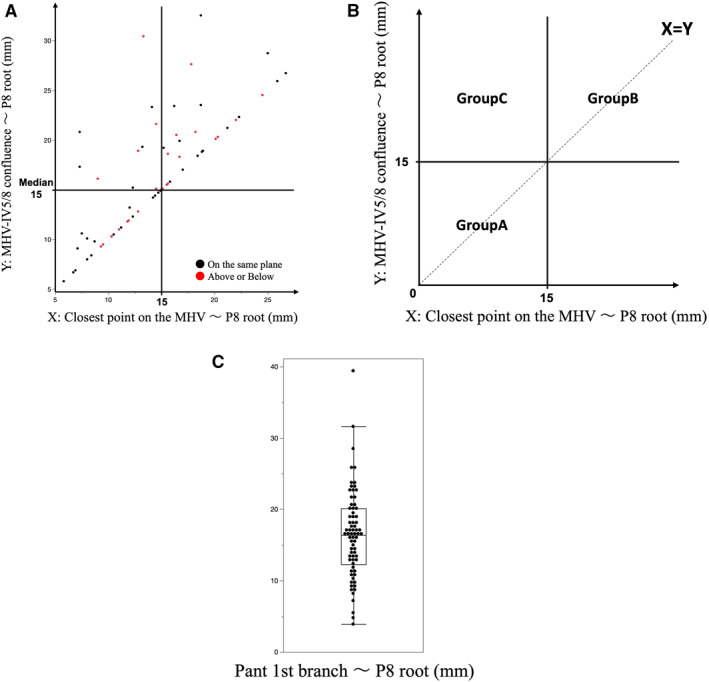
Scatter plot illustrating the positional relationships between the P8 root and key anatomical landmarks. (A) The plot includes 65 cases, excluding 8 cases where the MHV–IV5/8 confluence was not identified. The horizontal axis (*X*) represents the shortest distance from the MHV to the P8 root, and the vertical axis (*Y*) represents the distance from the MHV–IV5/8 confluence to the P8 root. Black circles indicate cases in which the P8 root aligns with the MHV–RHV plane, whereas red circles indicate misalignment. The median value of 15 mm for X divides the plot into three groups, with the *X* = *Y* line representing cases in which the shortest distance from the P8 root to the MHV matches the MHV–IV5/8 confluence. (B) Classification of cases into three groups based on the median value of 15 mm for *X* and *Y*: Group A (*X* ≤ 15 mm and *Y* ≤ 15 mm), Group B (*X* > 15 mm and *Y* > 15 mm), and Group C (*X* ≤ 15 mm and *Y* > 15 mm). (C) Distance from the first branch of the Pant to the P8 root (mm). IV5/8, Intersegmental vein between segments 5 and 8; MHV, middle hepatic vein; P8, Portal vein of segment 8; Pant, right anterior portal vein.

The presence of the IV5/8 and its drainage pattern are prerequisites for its use as a landmark in HVGA. Equality of the shortest distance from the P8 root to the MHV (*X*) and the distance from the MHV–IV5/8 confluence to the P8 root (*Y*) (*X* = *Y*), or short values of *X* and *Y*, indicates that the IV5/8 or MHV can serve as reliable landmarks for identifying the G8 root in HVGA. To extract cases suitable for HVGA based on these factors, cases were classified into Groups A, B, and C (Figure 4A,B). The MHV–RHV plane and the P8 root are not always located on the same plane, which can increase the difficulty of HVGA; therefore, this spatial relationship was also analyzed. The distance from the hepatic hilum to the P8 root was assessed as an indicator of the technical difficulty of EHGA (Figure 4C). Patterns of right anterior portal ramification were also recorded as background data but were not analyzed for their impact on approach selection.

### 3.1 Right Anterior Portal Ramification and Venous Drainage Patterns

Among the 73 cases, the right anterior portal vein ramification patterns of the Pant were cranio‐caudal in 28 cases (38.3%), ventro‐dorsal in 17 cases (23.3%), trifurcation in 18 cases (24.7%), and unclassifiable in 10 cases (13.7%). The IV5/8 branch was not identified in 7 cases (9.6%). IV5/8 drained into the MHV in 53 cases (72.6%), the RHV in 1 case (1.4%), and both the MHV and RHV in 12 cases (16.4%; Table 1).

**TABLE 1:** Anatomical variations in donor candidates.

Variables	Donor candidates *n* = 73
Right anterior portal vein ramification pattern; *n* (%)	
Cranio‐caudal type	28 (38.3)
Ventro‐dorsal type	17 (23.3)
Trifurcation type	18 (24.7)
Unclassifiable	10 (13.7)
Drainage site of the IV5/8; *n* (%)	
Not identified	7 (9.6)
Single MHV	53 (72.6)
Single RHV	1 (1.4)
Both MHV and RHV	12 (16.4)
P8 position relative to the MHV‐RHV plane; *n* (%)	
Above	22 (30.1)
On the same plane	45 (61.6)
Below	6 (8.2)
Distance (mm); median (range)	
Closest point on the MHV to the root of P8 (*X*)	14.5 (5.1–28.9)
MHV–IV5/8 confluence to the root of P8 (*Y*)^a^	15.6 (5.8–32.5)
Pant 1st branch to the root of P8	16.4 (3.9–39.4)

Abbreviations: IV5/8, intersegmental vein between segment 5 and 8; MHV, middle hepatic vein; MHV–IV5/8 confluence, confluence of IV5/8 and MHV; P8, portal vein of segment 8; Pant, right anterior portal vein; RHV, right hepatic vein.

^a^ Analysis was conducted on 65 patients after excluding 8 patients with unidentifiable IV5/8 or those draining only into the RHV.

### 3.2 Factors Indicating the Position of the G8 Root

The position of the P8 root relative to the MHV–RHV plane was “above” in 22 cases (30.1%), “on the same plane” in 45 cases (61.6%), and “below” in 6 cases (8.2%). Among all 73 cases, the median (range) shortest distance between the MHV and the root of P8 (*X*) was 14.5 mm (5.1–28.9). The median distance between the MHV–IV5/8 confluence and the root of P8 (*Y*) was 15.6 mm (5.8–32.5) in the 65 cases where the IV5/8 drained into the MHV. The median distance from the first branch of the Pant to the root of P8 was 16.4 mm (3.9–39.4) (Table 1).

Figure 4A presents a scatter plot of 65 cases, excluding 8 cases where the MHV–IV5/8 confluence was not identified. The horizontal axis represents *X*, and the vertical axis represents *Y*. Black circles indicate cases where the P8 root lies on the MHV–RHV plane, whereas red circles indicate deviations from this plane. The median value of 15 mm for *X*, the shortest distance from the MHV, was used to divide the plot into sections along the *X*‐ and *Y*‐axes. Based on *X* and Y values, cases were classified into three groups. Group A had both *X* and *Y* ≤ 15 mm, Group B had both *X* and *Y* > 15 mm, and Group C had *X* ≤ 15 mm but *Y* > 15 mm. Cases where the P8 root was at the same distance from the MHV and the MHV–IV5/8 confluence were plotted on the *X* = *Y* line (Figure 4B). Among the 65 cases, Group A included 27 cases (41.5%), Group B 28 cases (43.1%), and Group C 10 cases (15.3%). *X* = *Y* was observed in 39 cases (60.0%), whereas *X* ≠ Y was found in 26 cases (40.0%). Group A with *X* = *Y* included 22 cases (33.8%), and Group B with *X* = *Y* had 17 cases (26.1%). Notably, only 15 cases (23.0%) were classified as Group A with *X* = *Y* and black circles.

The median distance from the first branch of the Pant to the root of P8 was 16.4 mm, with a wide range of 3.9–39.4 mm (Figure 4C).

### 3.3 Hybrid Glissonean Approach: A Novel Technique for Anatomical Segmentectomy 8

**FIGURE 5:**
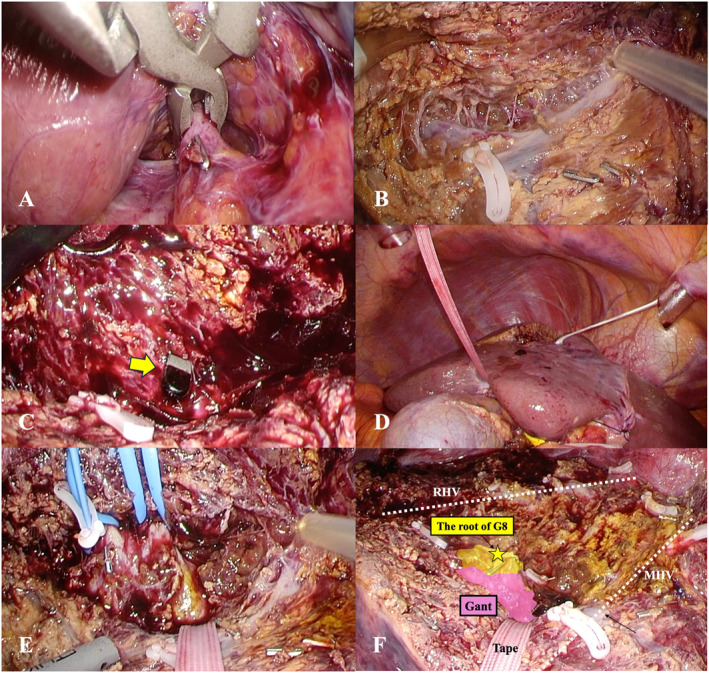
Intraoperative findings of the hybrid Glissonean approach. (A) The Gant is minimally exposed and clamped using detachable clamp forceps. (B) Liver parenchymal transection and exposure of the middle hepatic vein. (C) The Endo Mini‐Retract (Medtronic, USA) is inserted from the hepatic hilum to the transection plane, along the inner side of the Gant. (D) Taping is performed to guide liver parenchyma transection. (E) The G8 roots are identified by tracing the Gant. (F) After specimen extraction. The Gant surface and the G8 root are recognized from the transection plane. G8, Glissonean pedicle of segment 8; Gant, anterior Glissonean pedicle; MHV, middle hepatic vein.

The HYBA technique for anatomical segmentectomy 8, developed at our institution, is outlined as follows: After dissecting the falciform ligament, the roots of the MHV and RHV are identified. The right lobe is partially retracted to expose the surface of S8. To prepare for the Pringle maneuver, the hepatoduodenal ligament is taped. Subsequently, the gallbladder plate is taped extrahepatically, and cholecystectomy is performed. The gallbladder plate is then dissected, and the Gant is partially exposed from the hepatic hilum for blunt clamping. At this stage, minimal detachment of the Gant is performed without encircling or taping. The midplane is marked along the demarcation line, which is determined by temporally clamping the Gant with detachable clamp forceps (Figure 5A). Liver transection is performed along the midplane under the Pringle maneuver, guided by the demarcation line, to expose the MHV. Once the MHV is sufficiently exposed (Figure 5B), forceps are used to probe from the inside of the Gant toward the transection plane. The movement of the liver parenchyma indicates the directional orientation of the Gant surface relative to the transection plane, a technique referred to as the “contact test.” The forceps is inserted from the hepatic hilum to the transection plane, along the inner side of the Gant for taping (Figure 5C). This tape is used as a guide to identify the Gant and the G8 root from the transection plane (Figure 5D). By tracing the Gant, the G8 roots are identified and resected (Figure 5E). The boundary between segments 5 and 8, as well as the anterior and posterior sections, is marked using indocyanine green after blocking the inflow. Liver transection continues to expose the RHV from the medial and cranial sides. Finally, the remaining liver parenchyma is lifted with tape for specimen extraction. Identifying the Gant surface from the transection plane enables precise G8 root detachment (Figure 5F). This technique is further demonstrated in the Video 1.

**VIDEO 1:** Surgical technique for the hybrid Glissonean approach in anatomical segmentectomy 8. To view this video in the full‐text HTML version of the article, please visit https://onlinelibrary.wiley.com/doi/10.1002/wjs.70129.

## 4 Discussion

This study demonstrated anatomical variations in the positional relationships of the G8 root with key landmarks such as the MHV, RHV, IV5/8, and hepatic hilum. Some cases exhibited misalignment of the P8 root with the MHV‐RHV plane or undetectable IV5/8, with highly variable distances from the MHV‐IV5/8 confluence, MHV, and hepatic hilum to the P8 root. These anatomical factors inform the selection of an optimal surgical approach for anatomical segmentectomy 8. Notably, cases in which the G8 root could be readily identified from only the hepatic vein side or hilar side were limited; in such cases, HYBA may serve as a viable option.

IV5/8 is an important anatomical landmark for identifying the G8 root from the midplane in HVGA [6]. In this study, the IV5/8 was not identified in 9.6% of cases. In comparison, Hang et al. reported a higher incidence of IV5/8 absence (24.6%) based on CT scans of 57 patients with liver tumors [14]. This discrepancy may be attributed to the use of high‐quality CT images of healthy donors with normal livers in our study, which likely enabled more precise vascular visualization. These findings suggest that in clinical settings involving tumors or background liver disease, preoperative identification of the IV5/8 may be difficult in > 10% of cases.

Even when the IV5/8 was present, the distance between the MHV‐IV5/8 confluence and the P8 root (*Y*) showed wide variability (5.8–32.5 mm), with the MHV‐IV5/8 confluence being closest to the P8 root on the midplane (*X* = *Y*) in only 54.8% of cases. These findings indicate that the IV5/8 is an effective landmark in only a limited number of cases, making HVGA less suitable in such situations. In addition, HVGA identifies the G8 root by exposing the MHV and RHV from their roots and using the MHV–RHV plane as a guide [4]; therefore, our anatomical analysis demonstrated that the P8 root was located above this plane in 30.1% of cases, on the same plane in 61.6%, and below it in 8.2%. These findings indicate that HVGA may result in disorientation and blind dissection in cases where the P8 root is not aligned with the MHV–RHV plane. 3D variations further complicate localization of the G8 root. In contrast, HYBA enables identification of the G8 root even when it is distant from the IV5/8 confluence or when it is not located on the MHV–RHV plane by using the contact test and taping. Additionally, lifting the liver parenchyma with tape helps prevent injury to the dorsal portion of the Glisson’s capsule.

Although EHGA enables accurate identification of the G8 root, this approach becomes challenging when the root is located far from the hepatic hilum. In this study, the distance from the first branch of the Gant to the P8 root varied widely, ranging 3.9–39.4 mm. When the G8 root was positioned deeper, a wider dissection of the hepatic hilum was required, and there was a need to tape the G5, which is typically untouched in other approaches. EHGA has been associated with an increased risk of bile leakage [15], particularly in patients with liver cirrhosis, due to tissue fragility [16]. In HYBA, taping and transection of the G8 root are performed from the transection plane. The Gant is exposed from the hepatic hilum with minimal dissection, which reduces the risk of bile leakage.

Another advantage of HYBA is that it obtains the demarcation line of the anatomical midplane by clamping the Gant. In HVGA, liver transection is guided by intraoperative ultrasonography to identify the MHV and IV5/8; however, this differs from the anatomical midplane defined by the portal territory. For anatomical segmentectomy 8, clamping the right Glissonean pedicle has been reported to identify the anatomical midplane by the demarcation line [17, 18]. However, unless the Gant is exposed from the hepatic hilum, clear identification of both the Gant and the G8 root from the transection plane is challenging.

At our institution, the surgical approach, that is, EHGA, HVGA, or HYBA, is selected based on anatomical characteristics of the G8 root as assessed by preoperative MDCT. Factors such as the depth of the G8 root from the hepatic hilum and its relationship with surrounding landmarks may influence the feasibility of each approach. EHGA is suitable when the G8 root is close to and readily accessible from the hepatic hilum, whereas HYBA is preferred when the G8 root is located deeper. HVGA is suitable when the IV5/8 is present and drains into the MHV, with such cases belonging to Group A (27 cases; 36.9%), where the G8 root is located relatively close to the midplane. Moreover, when the closest point on the MHV to the G8 root matches the MHV‐IV5/8 confluence (*X* = *Y*), and the G8 root is aligned with the MHV‐RHV plane (black circles in Figure 4A,B), HVGA enables easier identification of the G8 root. Conversely, when the G8 root is distant from the MHV or MHV‐IV5/8 confluence and difficult to identify (Group B or C), particularly in cases with *X* ≠ *Y* and red circles, HVGA is avoided. In such cases, HYBA is preferred, as this approach enables identification of the G8 root based on the direction of the Gant exposed from the hepatic hilum. EHGA is selected when the G8 root is close to the hepatic hilum. In patients with cirrhosis, EHGA was avoided due to the increased risk of postoperative bile leakage [16], and HVGA is prioritized to minimize complications. Although tumor location may also influence approach selection in clinical practice, this aspect was not directly evaluated in the present study.

Among these indications, 19 anatomical segmentectomies for segment 8 were performed at our institution between 2016 and 2024, including 3, 12, and 4 cases of EHGA, HVGA, and HYBA cases, respectively (Table 2). A comparative anatomical analysis revealed that the distance from the hepatic hilum to the P8 root was significantly shorter in the EHGA group (median, 6.0 mm) than in the HVGA group (median, 19.4 mm; *p* = 0.001). Notably, 50% of the HYBA cases involved patients in whom the IV5/8 could not be identified preoperatively. In the HVGA group, the IV5/8 could not be identified in one case (8.3%), which involved a patient with liver cirrhosis (F4). One case of postoperative bile leakage was observed in the EHGA group. However, no significant differences were noted in operative time or blood loss among the three approaches. This study did not compare the approaches for superiority or inferiority but presented their respective anatomical considerations and clinical applications. In the future, surgeons performing anatomical segmentectomy of segment 8 should consider these anatomical factors, which can be easily measured using standard CT alone when selecting the most appropriate approach (Table 2).

**TABLE 2:** Anatomical and clinical features by approach for segmentectomy 8.

Approach	No.	Lap/robot	Anatomical factors						Intraoperative findings		Pathological findings			Postoperative findings		
			P8 position relative to the MHV‐RHV plane	Drainage site of the IV5/8	Group	*X* (mm)	*Y* (mm)	Pant 1st branch 〜P8 root (mm)	Operation time (min)	Blood loss (mL)	*F*	Tumor size (mm)	Resection margin	Complication CD ≥ 3a	POBL (ISGLS)	Postoperative hospital stays (day)
EHGA	1	Lap	On the same plane	MHV and RHV	B	16.3	16.3	13.7	445	100	1	24	R0	—	—	10
EHGA	2	Lap	On the same plane	Single MHV	B	19.3	19.3	6.0	486	50	1	52	R0	POBL	Grade B	18
EHGA	3	Lap	Above	Single MHV	A	12.0	12.0	5.3	445	5	2	30	R0	—	—	14
Median	*n* = 3					16.3 (12.0–19.3)	16.3 (12.0–19.3)	6.0 (5.3–13.7)*	445 (445–486)	50 (5–100)		30 (24–52)				14 (10–18)
HVGA	1	Lap	On the same plane	Single MHV	C	6.8	24.6	19.5	260	5	0	33	R1	—	—	9
HVGA	2	Lap	On the same plane	Single MHV	C	11.0	18.3	22.4	219	10	0	32	R0	—	—	8
HVGA	3	Lap	Above	MHV and RHV	A	13.9	13.9	19.8	339	50	2	30	R0	—	—	10
HVGA	4	Lap	On the same plane	MHV and RHV	A	12.0	12.0	20.6	496	250	0	31	R0	Ileus	—	22
HVGA	5	Lap	On the same plane	MHV and RHV	B	25.4	25.4	19.2	659	150	0	21	R0	—	—	9
HVGA	6	Lap	Below	Single MHV	B	20.2	20.2	14.7	371	5	1	32	R0	Respiratory failure	—	14
HVGA	7	Lap	Above	Single MHV	C	12.7	16.7	27.7	263	5	0	35	R0	—	—	12
HVGA	8	Lap	On the same plane	Single MHV	B	15.1	21.2	15.9	491	50	0	13	R0	—	—	42
HVGA	9	Lap	On the same plane	Single MHV	A	9.7	9.7	15.2	414	50	2	25	R0	—	—	23
HVGA	10	Lap	On the same plane	MHV and RHV	A	10.8	10.8	15.2	447	100	0	15	R0	—	—	11
HVGA	11	Lap	On the same plane	—	—	13.9	—	21.0	558	95	4	16	R0	—	—	10
HVGA	12	Robot	On the same plane	MHV and RHV	A	8.0	8.0	14.6	611	5	0	30	R0	—	—	7
Median	*n* = 12					12.3 (6.8–25.4)	16.7 (8.0–25.4)	19.4 (14.6–27.7)*	430 (219–659)	50 (5–250)		30 (13–35)				10 (7–42)
HYBA	1	Lap	Below	—	—	15.3	—	16.0	395	5	3	34	R0	Pleural effusion	—	16
HYBA	2	Lap	Above	Single MHV	B	22.6	22.6	14.3	493	100	0	43	R0	—	—	10
HYBA	3	Lap	On the same plane	—	—	12.6	—	27.2	396	150	2	36	R0	—	—	11
HYBA	4	Robot	On the same plane	Single MHV	B	20.4	23.4	5.8	347	5	0	31	R0	—	—	10
Median	*n* = 4					17.9 (12.6–22.6)	23.0 (22.6–23.4)	15.2 (5.8–27.2)	395 (347–493)	52 (5–150)		35 (31–43)				10 (10–16)

*Note: X* = Shortest distance between MHV and the root of P8 (mm), *Y* = Distance between IV5/8 and the root of P8 (mm), distance from the Pant 1st branch to the root of P8 (mm).

Abbreviations: CD, Clavien–Dindo classification; EHGA, extrahepatic Glissonean approach; F, fibrosis; HVGA, hepatic vein‐guided approach; HYBA, hybrid Glissonean approach; ISGLS, International Study Group of Liver Surgery; IV5/8, intersegmental vein between segment 5 and 8; Lap, laparoscopic; MHV, middle hepatic vein; MHV‐IV5/8 confluence, confluence of IV5/8 and MHV; P8, portal vein of segment 8; Pant, right anterior portal vein; POBL, postoperative bile leakage; RHV, right hepatic vein; Robot, robotic.

^*^ *p* = 0.001.

This study had some limitations. The Glissonean pedicle could not be directly visualized on preoperative MDCT simulations; therefore, the portal vein was used as the assessment target. As described by Sugioka et al. [9], the position of the Gant root is defined by anatomical landmarks unrelated to the portal vein. To address the positional discrepancy between the Gant and Pant roots, the distance to the P8 root was defined as the distance from the first branch of the Pant. Nevertheless, this approach may still have resulted in discrepancies between the preoperative simulation and intraoperative findings. Additionally, the small number of cases limits the ability of the study to compare surgical outcomes between the three approaches—HVGA, EHGA, and HYBA.

We evaluated the anatomical factors essential for anatomical segmentectomy 8, focusing on the position of the G8 root. Significant variations were observed in the positional relationships with key landmarks, including the MHV, RHV, IV5/8, and hepatic hilum. Preoperative MDCT evaluation of these relationships is crucial for selecting an appropriate surgical approach. HYBA is a viable option in cases where conventional approaches are limited. Further studies are required to evaluate each approach in terms of oncological safety, postoperative complications, and long‐term survival. Furthermore, the compatibility of each approach with minimally invasive liver surgery and the potential benefits of each approach should be evaluated.

## Author Contributions

**Yosuke Uematsu:** conceptualization, data curation, formal analysis, investigation, visualization, writing – original draft, and writing – review and editing. **Yuta Abe:** conceptualization, methodology, project administration, supervision, and writing – review and editing. **Yutaka Nakano:** writing – review and editing. **Minoru Kitago:** writing – review and editing. **Yasushi Hasegawa:** writing – review and editing. **Shutaro Hori:** writing – review and editing. **Masayuki Tanaka:** writing – review and editing. **Minoru Yamada:** investigation, data curation, visualization, and writing – review and editing. **Masahiro Jinzaki:** investigation, data curation, visualization, and writing – review and editing. **Yuko Kitagawa:** supervision, project administration, funding acquisition, and writing – review and editing. All authors reviewed and approved the final manuscript.

## Ethics Statement

This study was approved by the Ethics Committee of Keio University School of Medicine (approval number: 20120443).

## Consent

The requirement for informed consent was waived owing to the retrospective study design.

## Conflicts of Interest

Yuko Kitagawa has received institutional research grants from Asahi Kasei Pharma Corporation, Taiho Pharmaceutical Co. Ltd., Chugai Pharmaceutical Co. Ltd., Kaken Pharmaceutical Co. Ltd., Kowa Company Ltd., Otsuka Pharmaceutical Co. Ltd., Kyowa Hakko Kirin Co. Ltd., Sumitomo Pharma Co. Ltd., and Terumo Corporation. He has also received personal fees from Asahi Kasei Pharma Corporation, Taiho Pharmaceutical Co. Ltd., Chugai Pharmaceutical Co. Ltd., Kaken Pharmaceutical Co. Ltd., Kowa Company Ltd., Ethicon Inc., Ono Pharmaceutical Co. Ltd., Otsuka Pharmaceutical Factory Inc., Olympus Corporation, Cardinal Health K.K., Shionogi & Co. Ltd., Bristol‐Myers Squibb K.K., MSD K.K., ASKA Pharmaceutical Co. Ltd., Miyarisan Pharmaceutical Co. Ltd., Toray Industries Inc., Daiichi Sankyo Company, Limited, Chugai Foundation for Innovative Drug Discovery Science, Nippon Kayaku Co. Ltd., EA Pharma Co. Ltd., Intuitive Surgical G.K., Takeda Pharmaceutical Company Limited, Sysmex Corporation, Tsumura & Co., AI Medical Service Inc., and Eisai Co. Ltd. Additionally, he participates in joint research with Sysmex Corporation and Medicaroid Corporation, and serves as Editor‐in‐Chief of Annals of Gastroenterological Surgery, the official journal of the Japanese Society of Gastroenterological Surgery. Drs. Yosuke Uematsu, Yuta Abe, Yutaka Nakano, Minoru Kitago, Yasushi Hasegawa, Shutaro Hori, Masayuki Tanaka, Minoru Yamada, and Masahiro Jinzaki have no conflicts of interest or financial ties to disclose.

## Data Availability

The data that support the findings of this study are available from the corresponding author upon reasonable request.

## References

[1] G. Wakabayashi , D. Cherqui , D. A. Geller , et al., “The Tokyo 2020 Terminology of Liver Anatomy and Resections: Updates of the Brisbane 2000 System,” Journal of Hepato‐Biliary‐Pancreatic Sciences 29, no. 1 (2022): 6–15, 10.1002/jhbp.1091. 34866349

[2] T. Kobayashi , T. Ebata , Y. Yokoyama , et al., “Study on the Segmentation of the Right Anterior Sector of the Liver,” Surgery 161, no. 6 (2017): 1536–1542, 10.1016/j.surg.2016.12.020. 28126253

[3] N. Gotohda , D. Cherqui , D. A. Geller , et al., “Expert Consensus Guidelines: How to Safely Perform Minimally Invasive Anatomic Liver Resection,” Journal of Hepato‐Biliary‐Pancreatic Sciences 29, no. 1 (2022): 16–32, 10.1002/jhbp.1079. 34779150

[4] K. Monden , F. Alconchel , G. Berardi , et al., “Landmarks and Techniques to Perform Minimally Invasive Liver Surgery: A Systematic Review With a Focus on Hepatic Outflow,” Journal of Hepato‐Biliary‐Pancreatic Sciences 29, no. 1 (2022): 66–81, 10.1002/jhbp.898. 33475254

[5] M. Morimoto , F. Tomassini , G. Berardi , et al., “Glissonean Approach for Hepatic Inflow Control in Minimally Invasive Anatomic Liver Resection,” Journal of Hepato‐Biliary‐Pancreatic Sciences 29, no. 1 (2022): 51–65, 10.1002/jhbp.908. 33528877

[6] Y. Ome , G. Honda , M. Doi , J. Muto , and Y. Seyama , “Laparoscopic Anatomic Liver Resection of Segment 8 Using Intrahepatic Glissonean Approach,” Journal of the American College of Surgeons 230, no. 3 (2020): e13–e20, 10.1016/j.jamcollsurg.2019.11.008. 31783094

[7] K. Monden , H. Sadamori , M. Hioki , S. Ohno , and N. Takakura , “Laparoscopic Anatomic Liver Resection of the Dorsal Part of Segment 8 Using an Hepatic Vein‐Guided Approach,” Annals of Surgical Oncology 29, no. 1 (2022): 341, 10.1245/s10434-021-10488-y. 34302229

[8] K. Takasaki , S. Kobayashi , S. Tanaka , A. Saito , M. Yamamoto , and F. Hanyu , “Highly Anatomically Systematized Hepatic Resection With Glissonean Sheath Code Transection at the Hepatic Hilus,” International Surgery 75, no. 2 (1990): 73–77. 2166006

[9] A. Sugioka , Y. Kato , and Y. Tanahashi , “Systematic Extrahepatic Glissonean Pedicle Isolation for Anatomical Liver Resection Based on Laennec’s Capsule: Proposal of a Novel Comprehensive Surgical Anatomy of the Liver,” Journal of Hepato‐Biliary‐Pancreatic Sciences 24, no. 1 (2017): 17–23, 10.1002/jhbp.410. 28156078 PMC5299460

[10] C. Xiang , Z. Liu , J. Dong , K. Sano , and M. Makuuchi , “Precise Anatomical Resection of the Ventral Part of Segment VIII,” International Journal of Surgery Case Reports 5, no. 12 (2014): 924–926, 10.1016/j.ijscr.2014.10.041. 25460437 PMC4276076

[11] K. Monden , H. Sadamori , T. Iwasaki , M. Hioki , and N. Takakura , “Hepatic Vein‐Guided Approach in Laparoscopic Anatomic Liver Resection of the Ventral and Dorsal Parts of Segment 8,” Journal of Personalized Medicine 13, no. 6 (2023): 1007, 10.3390/jpm13061007. 37373996 PMC10305108

[12] Y. Kato , A. Sugioka , M. Kojima , et al., “Laparoscopic Isolated Liver Segmentectomy 8 for Malignant Tumors: Techniques and Comparison of Surgical Results With the Open Approach Using a Propensity Score‐Matched Study,” Langenbeck’s Archives of Surgery 407, no. 7 (2022): 2881–2892, 10.1007/s00423-022-02673-8. 36102966

[13] L. R. Koetzier , D. Mastrodicasa , T. P. Szczykutowicz , et al., “Deep Learning Image Reconstruction for CT: Technical Principles and Clinical Prospects,” Radiology 306, no. 3 (2023): e221257, 10.1148/radiol.221257. 36719287 PMC9968777

[14] H. Li , Z. Shao , Z. Song , M. Han , Z. Cheng , and X. Song , “Study of the Intersegmental Veins Between S5 and S8 Based on 3D Reconstruction,” Journal of Gastrointestinal Surgery 27, no. 10 (2023): 2085–2091, 10.1007/s11605-023-05766-x. 37433951

[15] Y. I. Yamashita , H. Yamamoto , H. Miyata , et al., “Risk Factors for Bile Leakage: Latest Analysis of 10 102 Hepatectomies for Hepatocellular Carcinoma From the Japanese National Clinical Database,” Journal of Hepato‐Biliary‐Pancreatic Sciences 28, no. 7 (2021): 556–562, 10.1002/jhbp.827. 32897639

[16] K. Hayashi , Y. Abe , M. Shinoda , et al., “Clinical Impact of Intraoperative Bile Leakage During Laparoscopic Liver Resection,” Surgical Endoscopy 35, no. 8 (2021): 4134–4142, 10.1007/s00464-020-07880-2. 32780232

[17] J. H. Kim and H. Kim , “Pure Laparoscopic Anatomic Resection of the Segment 8 Ventral Area Using the Transfissural Glissonean Approach,” Annals of Surgical Oncology 26, no. 13 (2019): 4608–4609, 10.1245/s10434-019-07852-4. 31583544

[18] J. H. Kim , “Laparoscopic Anatomical Segmentectomy Using the Transfissural Glissonean Approach,” Langenbeck’s Archives of Surgery 405, no. 3 (2020): 365–372, 10.1007/s00423-020-01889-w. 32388715

